# Understanding Burnout in Forensic Medicine and the Interaction of Job Satisfaction and Unconditional Self-Acceptance: A Cross-Sectional Study

**DOI:** 10.3390/healthcare13101169

**Published:** 2025-05-16

**Authors:** Lilioara-Alexandra Oprinca-Muja, Adrian-Nicolae Cristian, Elena Topîrcean, Alina Cristian, Marius Florentin Popa, Horațiu Paul Domnariu, Diter Atasie, George-Călin Oprinca, Silviu Morar

**Affiliations:** 1 Faculty of Medicine, Lucian Blaga University of Sibiu, 550169 Sibiu, Romania; lilioaraalexandra.muja@ulbsibiu.ro (L.-A.O.-M.); elena.topircean@ulbsibiu.ro (E.T.); alina.cristian@ulbsibiu.ro (A.C.); horatiupaul.domnariu@ulbsibiu.ro (H.P.D.); atasie.diter@ulbsibiu.ro (D.A.); georgecalin.oprinca@ulbsibiu.ro (G.-C.O.); silviu.morar@ulbsibiu.ro (S.M.); 2 Faculty of Medicine, Ovidius University of Constanța, 900527 Constanța, Romania; marius_popa2005@yahoo.com

**Keywords:** burnout, job satisfaction, unconditional self-acceptance, forensic medicine, Maslach Burnout Inventory, mediation analysis

## Abstract

Background and Objectives: Burnout syndrome is increasingly recognized as a significant occupational hazard among forensic medicine professionals, a population exposed to intense psychological stress and complex work demands. This study aimed to assess the prevalence of burnout, job satisfaction, and unconditional self-acceptance among forensic personnel in Romania and to explore potential predictors and mediators of burnout within this context. Materials and Methods: A cross-sectional survey was conducted among 153 forensic medicine professionals from 31 counties across Romania. Participants completed a battery of standardized instruments, including the Maslach Burnout Inventory—General Survey (MBI-GS), the Job Satisfaction Survey (JSS), and the Unconditional Self-Acceptance Questionnaire (USAQ). Cut-off scores for burnout subscales were established using the 75th percentile. Data were analyzed to identify demographic, occupational, and psychological correlates of burnout. Descriptive statistics were used to summarize sample characteristics and burnout prevalence. Group comparisons were made across demographic and professional categories. A mediation model was tested to evaluate whether unconditional self-acceptance mediated the relationship between job satisfaction and burnout. We hypothesized that job satisfaction would be negatively associated with burnout symptoms and that unconditional self-acceptance would mediate this relationship, acting as a protective psychological factor. Results: Approximately a quarter of respondents met the criteria for high total burnout, emotional exhaustion, and professional inefficacy, while cynicism was present in a fifth of participants. Burnout was more prevalent among younger workers, forensic medicine residents, and those working in the capital. Job satisfaction was generally high, but lower in women, younger individuals, and residents. Nearly half of the participants reported low or very low self-acceptance. Mediation analysis revealed that unconditional self-acceptance partially mediated the relationship between job satisfaction and burnout, suggesting a protective psychological mechanism. Conclusions: This study highlights the considerable burden of burnout among forensic medicine professionals in Romania and identifies job dissatisfaction and low self-acceptance as key contributors. Findings underscore the need for targeted interventions aimed at enhancing job satisfaction and emotional resilience, particularly in vulnerable subgroups. Institutional efforts that support mental well-being, foster emotional coping, and improve organizational dynamics are essential to reduce burnout in this high-risk population.

## 1. Introduction

The concept of burnout as a psychological phenomenon resulting from work-related stress was introduced in the 1970s by Freudenberger, with further development by Maslach in the 1980s, who solidified the term through her research on human service workers and their responses to stressful situations [1]. Freudenberger initially observed signs of psychological distress among workers in a detoxification clinic in the 1960s. He later described these symptoms in the 1970s, coining the term “burn-out” to define a state of failure in which an individual becomes physically and emotionally depleted due to excessive demands on energy, strength, or resources [2]. Christina Maslach and her colleagues contributed significantly to the conceptualization and measurement of burnout by developing the Maslach Burnout Inventory (MBI), which remains one of the most widely used tools for assessing burnout today [3].

Burnout is generally understood as a psychological state of exhaustion resulting from prolonged exposure to work-related stressors, combined with an insufficient ability to cope with these stressors [4]. The latest ICD-11 defines burnout as “a syndrome conceptualized as resulting from chronic workplace stress that has not been successfully managed”. It is classified as an occupational phenomenon, not a medical condition [5]. According to Maslach et al., the three core components of burnout are high emotional exhaustion, depersonalization (or cynicism), and low personal accomplishment [3]. Emotional exhaustion refers to feelings of being emotionally drained due to continuous interpersonal interactions and is considered the central dimension of burnout. Depersonalization arises as a consequence of emotional exhaustion, manifesting as negative or excessively detached attitudes toward others, particularly clients or colleagues. Finally, a lack of personal accomplishment reflects a diminished sense of success and efficacy in one’s professional role.

In the case of healthcare workers, particularly physicians, the demanding nature of their specialty and the high-risk environment in which they operate contribute significantly to burnout. Similarly to other organizational settings, excessive workload in physicians leads to stress-related psychological dysfunctions. This includes the burden of examining a large number of patients, long working hours with rigid schedules, overnight shifts, and extensive administrative responsibilities. Also, physicians may feel isolated within their team, struggle with collaboration, or perceive colleagues as incompetent, further exacerbating stress levels. Loneliness and poor social support are known contributors to emotional exhaustion and were significantly associated with low recognition and weak attachment to the organization [6]. Moreover, in some countries, the imbalance between effort and reward is particularly pronounced, with inadequate financial compensation and insufficient resources to practice medicine under optimal conditions. These challenges contribute to increased stress, emotional exhaustion, reduced personal accomplishment, and ultimately burnout [7]. For medical residents, additional stressors further elevate the risk of burnout. These include the emotional burden of managing severely ill or dying patients, as well as concerns about their professional future. Factors such as uncertainty regarding job placement, lack of job security in certain countries, the quality of medical training, and conflicts with senior physicians add to their psychological strain [8].

When examining stressors associated with burnout syndrome in healthcare workers, most studies have identified three primary categories that contribute to chronic stress. These factors are socioeconomic, occupational, and psychological in nature [9]. Nurses in particular face a heightened risk, especially those exposed to heavy workloads, working multiple jobs, having limited experience in the field, or who are single [10]. Medical specialty is another consistent predictor of burnout, with surgical specialties being more affected than medical specialties. Additionally, healthcare workers in high-stress environments, such as intensive care units and oncology, exhibit higher burnout rates. This is primarily due to excessive workload, increased malpractice risks, and a lower probability of positive patient outcomes compared to other medical fields [11]. Regarding sociodemographic factors, both younger and more senior professionals are generally considered to have some degree of protection against burnout. Other protective occupational factors include job satisfaction, working primarily with adult patients, and maintaining a positive professional status within the workplace. In contrast, constant exposure to death and suffering, the persistent awareness that errors can have severe consequences, and the perception that some medical cases are impossible to manage are all occupational risk factors strongly associated with burnout [9]. Social stressors, such as a lack of psychological support from family or colleagues, further contribute to high levels of emotional exhaustion and depersonalization [12]. Among psychological factors, depression and anxiety are the most strongly associated with burnout [13].

Burnout syndrome in forensic medicine professionals has garnered increasing attention due to the unique psychological and occupational stressors inherent to their field. Unlike general healthcare settings, forensic workers are regularly exposed to death, trauma, and violence, often involving vulnerable populations such as children or victims of abuse. In this specific category of workers, the psychological strain can be attributed to both acute and chronic occupational stressors. Chronic stressors are compounded by persistent work-related demands, including staff shortages, insufficient institutional support, bureaucratic burdens, lack of recognition from leadership, inadequate financial compensation, poor communication, contamination risks, and lack of protective equipment [14,15]. Acute stressors can include cases involving child deaths, sexual abuse, highly decomposed remains, burned or fragmented corpses, as well as aggressive behaviors coming from detainees or psychiatric patients, all of which are associated with cumulative posttraumatic responses and increased risk of burnout [16,17,18]. Also, forensic medicine professionals are frequently exposed to the consequences of violence, including domestic violence [19], and repeated involvement in the documentation and investigation of such traumatic events is generative of chronic stress.

Several studies have highlighted that forensic healthcare workers, including forensic mental health nurses, doctors, and autopsy technicians, experience high levels of EE, moderate levels of DP, and low PA, as measured by the MBI [14,20,21]. In one study, 40.5% of forensic doctors reported high DP, 25% reported high EE, and 20.2% reported low PA [16]. Furthermore, autopsy technicians appear to be at greater risk of posttraumatic stress disorder (PTSD), exhibiting significantly higher levels of intrusive thoughts, physiological arousal, and avoidance behaviors compared to forensic physicians or residents [17]. A critical factor in mitigating burnout is the use of effective coping strategies. Forensic professionals who adopt maladaptive or dysfunctional coping mechanisms, such as avoidance, behavioral disengagement, or religious fatalism, tend to experience higher levels of EE and DP [14,21,22]. Also, alcohol consumption is observed among individuals working in morgue settings, serving as a widespread maladaptive coping mechanism in Romania [23] and further exacerbating burnout as well as other psychological issues. Conversely, adaptive coping strategies such as mindfulness and emotional self-awareness have demonstrated protective effects. Higher levels of mindfulness are associated with lower perceived stress and reduced burnout symptoms across all three MBI subscales in forensic medicine personnel [21].

Additionally, forensic workers employing active coping strategies such as problem-solving and positive reappraisal reported higher levels of well-being despite experiencing verbal or physical aggression from patients [18]. Another important factor that can disengage the link between chronic or acute stress and burnout is resilience. In forensic workers, resilience is positively associated with psychological well-being and negatively related to perceived work stress. Notably, workers with higher resilience report feeling less personally attacked or threatened in their roles [24]. Also, job satisfaction and emotional well-being are key moderating factors for burnout in general, but also in a forensic setting. Forensic professionals who report higher levels of job satisfaction exhibit lower EE and DP, even when confronted with high workloads or emotionally demanding cases [25,26,27]. In addition, personality traits and self-perception play a significant role in burnout vulnerability. High levels of neuroticism and low levels of extroversion are associated with greater EE and DP, whereas emotional regulation and unconditional self-acceptance appear to enhance resilience against professional fatigue and emotional detachment [22,26]. Despite the growing recognition of burnout among forensic medicine workers, institutional strategies for psychological support remain insufficient, and a majority of professionals report lacking a safe space to discuss mental health challenges within their workplace [28].

Studies on burnout in healthcare professions have also identified geographical patterns. Healthcare workers in hospitals across Southern and Eastern Europe tend to experience higher burnout levels compared to their counterparts in Scandinavian countries. Additionally, Eastern European healthcare professionals report greater burnout rates when working in public hospitals, whereas in Nordic countries, burnout appears to be more prevalent in the private sector [11]. These geographical variations are closely linked to differences in social and economic development, the strength of public healthcare infrastructure in Scandinavian countries, and the financial stability offered to medical professionals in the public sector. Many healthcare systems in Eastern Europe face persistent staff shortages, aging workforces, and increasing workloads, all of which exacerbate burnout risk. In teaching hospitals, the risk is amplified, as overburdened clinicians must also train future professionals [29]. There is an urgent need for systemic and individual-level interventions, including emotional support, task redistribution, resilience training, and improved work–life balance in these regions [29].

## 2. Materials and Methods

### 2.1. Design

This study aimed to provide insights into burnout syndrome among forensic medicine personnel and its correlation with job satisfaction and unconditional self-acceptance. A cross-sectional design was employed, along with convenience sampling. The study received ethics approval from the Universities Ethics Committee (approval 25/29 July 2024) and is in compliance with ethical standards for research. To ensure clarity and a well-structured presentation of results, we formulated several research questions based on the collected data:

- What is the overall burnout rate among forensic medicine personnel? Additionally, to what extent are emotional exhaustion, cynicism, and professional inefficacy present in these individuals, and at what levels?
- Are gender and age significant risk factors for burnout, emotional exhaustion, cynicism, and professional inefficacy?
- Does having children or being married serve as a protective factor or a risk factor for burnout among forensic medicine personnel?
- Which forensic professional category is most prone to burnout, emotional exhaustion, cynicism, and professional inefficacy?
- Does holding a leadership position increase the risk of burnout?
- Which Romanian region has the highest prevalence of burnout in forensic medicine personnel?
- What are the levels of job satisfaction among forensic medicine workers, and how do they vary by gender, age, marital status, parenthood status, job category, or leadership position?
- What are the levels of unconditional self-acceptance among forensic medicine workers, and how do they fluctuate based on gender, age, marital status, parenthood status, job category, or leadership position?
- Does low job satisfaction contribute to higher levels of burnout, emotional exhaustion, cynicism, and professional inefficacy?
- Is high unconditional self-acceptance a protective factor against burnout in forensic medicine workers?

The present study was conceptually grounded in the job demands–resources (JD-R) model, a widely used framework in occupational health psychology. This model posits that burnout develops when job demands such as workload or emotional exposure exceed job resources like autonomy and psychological support, leading to emotional exhaustion and disengagement. In this context, job satisfaction may act as a protective job resource, while unconditional self-acceptance may function as a personal resource that alleviates the effects of job strain. By applying the JD-R model to forensic medicine, a field marked by high emotional demands and limited psychological support, this study aimed to clarify how internal psychological factors may diminish the impact of job dissatisfaction on burnout.

Based on the job demands–resources model and prior findings in the field, we formulated the following hypotheses:

**H1:**  *A significant proportion of forensic medicine personnel experience elevated levels of burnout, including exhaustion, cynicism, and professional inefficacy.*

**H2:**  *Demographic factors such as younger age, male gender, being single, and not having children are associated with higher levels of burnout among forensic medicine professionals.*

**H3:**  *Professional factors, including being a resident in forensic medicine, lacking a leadership position, and working in certain geographical regions, are associated with an increased risk of burnout.*

**H4:**  *Forensic personnel reporting low levels of job satisfaction will exhibit significantly higher levels of burnout, including emotional exhaustion, depersonalization, and reduced personal accomplishment, compared to those with high job satisfaction.*

**H5:**  *Higher levels of unconditional self-acceptance are associated with lower levels of burnout among forensic medicine professionals.*

**H6:**  *Workers in leadership positions will report lower levels of burnout than those in non-leadership roles.*

**H7:**  *Unconditional self-acceptance mediates the relationship between job satisfaction and burnout such that higher self-acceptance reduces the negative impact of low job satisfaction on burnout symptoms.*

### 2.2. Participants

Forensic medicine personnel were invited to participate via institutional email invitations and through direct contact at national forensic medical conferences and department meetings. The survey was administered online through a secure, accessible, anonymous platform to ensure wide accessibility and participant comfort. To minimize self-report bias, participants were assured of the confidentiality and anonymity of their responses, and no identifying information was collected. The survey was accessed via a unique link or a QR code. Participation was voluntary and anonymous, and informed consent was obtained at the beginning of the questionnaire.

The study sample consisted of forensic medicine personnel from 31 county forensic medicine units across Romania. The inclusion criteria were as follows: age between 18 and 65 years; ability to understand instructions; active employment in forensic medicine during the study period; a minimum of six months of professional experience; and completion and submission of the full questionnaire with no missing responses.

The exclusion criteria were individuals on extended leave (e.g., medical leave, maternity leave, or sabbaticals) during the study period, part-time professionals with limited case exposure; incomplete questionnaire responses, and individuals with a history of mental health disorders that could interfere with their ability to provide accurate data (e.g., psychotic disorders, bipolar disorder).

Recruitment took place from August 2024 to September 2024, and no compensation was provided to participants. The period studied followed the peak disruptions of the COVID-19 pandemic, during which forensic medicine professionals in Romania had returned to relatively stable operational routines. The study also aimed to capture residual and potentially long-term effects of pandemic-related stressors on burnout and psychological well-being. A total of 205 forensic medicine professionals were invited to participate, and 153 completed the questionnaire, resulting in a response rate of 74.6%. The sample was predominantly female (62%). The average age of the participants was 44.39 years (SD = 10.38), with ages ranging from 24 to 63 years. A total of 136 participants resided in urban areas, while only 17 lived in rural areas. Approximately 54% (N = 82) of the workers had children in their care during the study period, and 71% (N = 109) were married. Among the remaining participants, 23 were unmarried, but in a serious relationship, 10 were single, 8 were divorced, and 3 were widowed. Regarding professional categories, the majority of participants were forensic medicine doctors (39%), while forensic medicine residents comprised 14% of the sample. Other staff members who had direct contact with the morgue (27%) included medical assistants (N = 33), autopsy technicians (N = 6), stretcher-bearers (N = 2), and drivers (N = 1). Notably, the low number of autopsy technicians who completed the questionnaire is due to the fact that most autopsy practitioners are also medical assistants, and they chose to report their profession as medical assistant in the questionnaire. Additional staff members (20%) included medical doctors from other specialties working in forensic medicine departments, such as psychiatrists, pathologists, and geneticists (N = 10), as well as biologists and chemists (N = 14). Auxiliary staff included medical registrars (N = 4) and cleaning attendants (N = 2). Most responses were from counties in the Transylvania region (N = 70), followed by the Moldova region (N = 20). The lowest number of participants came from the capital city, Bucharest, with only 8 respondents. Additionally, 14% of workers who completed the questionnaire held a leadership position.

### 2.3. Measures

Burnout was assessed using the Maslach Burnout Inventory—General Survey (MBI-GS) [30], a validated 16-item scale that covers three dimensions: exhaustion (5 items), cynicism (5 items), and professional inefficacy (6 items). Responses were recorded on a 5-point Likert scale ranging from 1 (very rarely) to 5 (very frequently). The inclusion of reverse-scored items aimed to enhance response variability and ensure consistency. Cronbach’s alpha for this scale in our sample was 0.909, indicating very good internal consistency. We calculated cut-off values for each subscale using the 75th percentile, as well as for total burnout scores. This threshold was selected in the absence of universally established norms and is consistent with prior research that uses percentile-based approaches to categorize high-risk groups. Respondents with scores above the thresholds were classified as severely emotionally exhausted, presenting elevated cynicism, or presenting high professional inefficacy, and were identified as having burnout syndrome.

The Job Satisfaction Survey (JSS) [31] assesses job satisfaction across multiple dimensions, including pay, promotion, supervision, benefits, and overall job satisfaction. The scale consists of 36 items, with responses measured on a 6-point Likert scale ranging from 1 (strongly disagree) to 6 (strongly agree). The JSS is designed to assess employees’ attitudes toward their work environment and various aspects of their job, including satisfaction with pay, promotion opportunities, supervision, fringe benefits, contingent rewards, operating conditions, coworkers, the nature of work, and communication. The scale has demonstrated good internal consistency, with Cronbach’s alpha of 0.911 indicating excellent reliability in our sample. Individuals were categorized based on their JSS scores as satisfied, ambivalent, or dissatisfied with their current employment.

The Unconditional Self-Acceptance Questionnaire (USAQ) [32] was used to measure individuals’ level of unconditional self-acceptance. The USAQ contains multiple items structured to assess how much a person accepts themselves regardless of their actions, thoughts, or feelings. Responses are measured on a Likert scale ranging from 1 (strongly disagree) to 5 (strongly agree). The scale includes both direct and reverse-scored items to ensure that respondents carefully consider their answers, reducing potential response biases. In our sample, the USAQ showed strong reliability, with Cronbach’s alpha coefficients indicating good internal consistency (0.760). Individuals were classified based on their responses into five categories: very strong, high, medium, low, or very low unconditional self-acceptance.

### 2.4. Analytical Plan

Data were entered in JASP (version 0.16.4) to be analyzed. There were no incomplete questionnaires and no missing data. Simple mediation analysis was performed with the SEM module. Job satisfaction was introduced as the predictor, unconditional acceptance was introduced as the mediator, and burnout level was introduced as the dependent variable. To assess the significance of the mediation effect, a percentile bootstrap with 1000 samples and a 95% confidence interval was estimated [33]. The effect is considered statistically significant if the confidence interval does not include zero.

The results of the multicollinearity analysis revealed that one of the independent variables, job satisfaction (VIF = 17.215), exhibited relatively high multicollinearity, suggesting that its strong correlation with other variables may affect the stability and reliability of the model’s estimates. In contrast, the unconditional acceptance variable had a VIF of 0.932, indicating no significant multicollinearity. While the assumption of linearity was largely met, as evidenced by scatterplots showing mostly linear relationships between variables, the homoscedasticity results suggested that the residuals were evenly distributed, supporting this assumption. However, given the identified multicollinearity issues, particularly with the job satisfaction variable, caution is advised when interpreting the findings. The high correlation of this variable with others could lead to unreliable coefficient estimates and inflated standard errors, potentially affecting the model’s precision.

We calculated the prevalence of emotional exhaustion, cynicism, professional inefficacy, and total burnout across our entire study cohort. Prevalence was analyzed and compared based on gender, age groups (under 30 years, 30–39 years, 40–49 years, and 50 years or older), and rural vs. urban living environment. Additionally, we compared burnout levels among married individuals, those in a relationship, and single individuals, with the latter category including those who were single, divorced, or widowed. We also examined differences between workers with children and those without children or with children over 18 years of age. Burnout prevalence was further compared across different professional categories, particularly between forensic medicine doctors, medical residents, and morgue staff, which included medical assistants, autopsy technicians, stretcher-bearers, and drivers. Additionally, we analyzed burnout levels in other staff members with no direct contact with bodies, such as pathologists, psychiatrists, geneticists, biologists, chemists, and auxiliary personnel. Finally, total burnout prevalence was compared between employees in leadership positions and those in non-leadership roles. The same statistical analyses and comparisons were applied to Job Satisfaction Survey (JSS) scores and Unconditional Self-Acceptance Questionnaire (USAQ) results to assess variations in job satisfaction and self-acceptance across different demographic and professional groups.

## 3. Results

### 3.1. Descriptive Analysis

**Figure 1:**
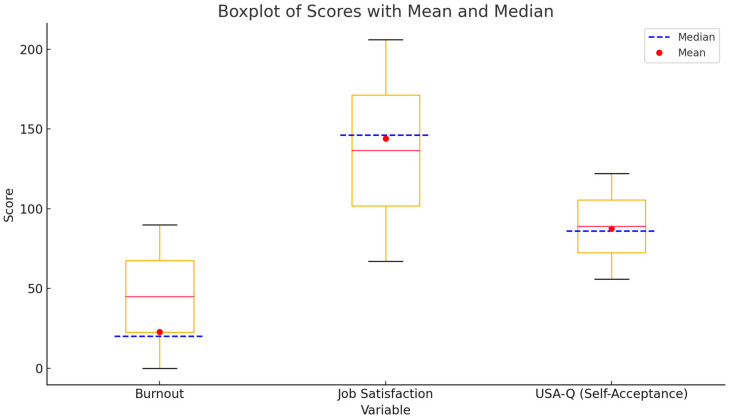
Descriptive statistics of burnout syndrome, job satisfaction, and unconditional self-acceptance in forensic medicine workers.

The results of the descriptive analysis (means, standard deviations, skewness) are given in Table 1, while the correlation matrix of the study variables is presented in Table 2. Pearson correlation coefficients were calculated to examine the relationships among job satisfaction, unconditional self-acceptance, and burnout dimensions. The variables present univariate normality, as none of them displays a skewness value greater than the recommended cut-off of 3 [34] (Figure 1).

Based on the calculation of the 75th percentile for emotional exhaustion, cynicism, professional inefficacy, and total burnout scores within our cohort, specific cut-off values were established for each subscale. The defined thresholds were: 10 for emotional exhaustion, 11 for cynicism, 11 for professional inefficacy, and 32 for total burnout.

Analysis of the MBI-GS results revealed that 22.87% of forensic medicine personnel were classified as experiencing high emotional exhaustion, 20.91% exhibited high levels of cynicism, and 23.52% demonstrated significant professional inefficacy. Additionally, 23.52% of participants had high total burnout scores. Notably, 17 individuals (representing 11.11% of the cohort) scored above the established cut-off values on all three subscales, indicating a high level of burnout across all dimensions.

**Figure 2:**
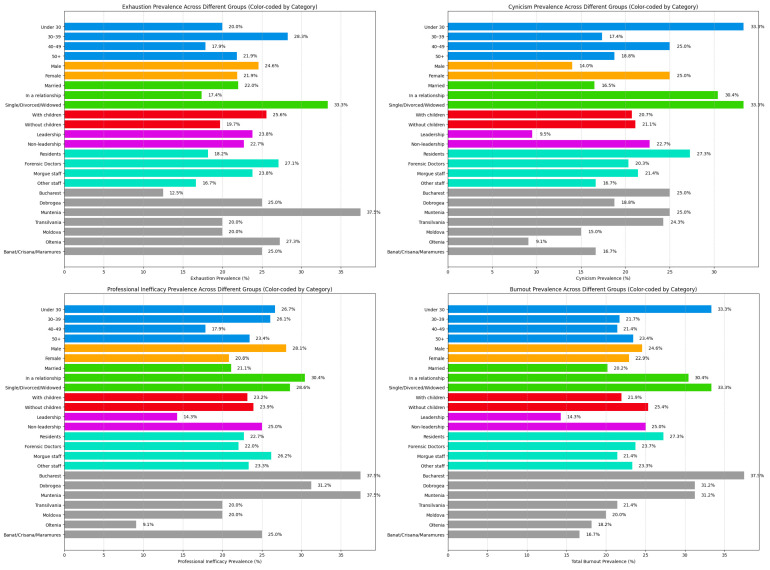
Exhaustion, cynicism, professional inefficacy, and total burnout score prevalence in different groups.

With respect to gender, results indicated a slightly higher prevalence of total burnout among male participants, with 24.56% scoring above the established threshold compared to 22.91% of female participants. The most pronounced difference in males was observed in the professional inefficacy subscale, where 28.07% of men reported high scores, in contrast to 20.83% of women. Conversely, cynicism appeared more prevalent among female forensic medicine workers, with 25% scoring above the cut-off value compared to only 14.03% of male participants. A slightly increased risk of emotional exhaustion was also noted among men (24.56%) relative to women (21.87%) (Figure 2).

Analysis of the results revealed that younger forensic medicine workers exhibited the highest rates of cynicism, professional inefficacy, and total burnout compared to their older counterparts. Specifically, 33.33% of individuals under the age of 30 reported high levels of total burnout. This was followed by 23.43% of workers aged over 50, 21.73% of those aged 30–39, and 21.42% of those aged 40–49, the latter two age groups showing similar levels of burnout. A prevalence of 33.33% was also observed on the cynicism subscale among workers under 30, followed by those aged 40–49, with 25% scoring above the cut-off. In terms of professional inefficacy, 26.66% of participants under 30 years of age scored highly, a rate very similar to the 30- to 39-year group (26.08%). The most professionally effective group appeared to be those aged 40–49 years, with only 17.85% scoring highly on professional inefficacy. The only subscale in which the under-30 age group did not have the highest prevalence was emotional exhaustion, where only 20% reported elevated scores. In contrast, 28.26% of workers aged 30–39 and 33.33% of those over 50 reported high levels of exhaustion (Figure 2).

Forensic medicine workers who were single, divorced, or widowed reported higher levels of emotional exhaustion, cynicism, and total burnout, with up to 33.33% scoring above the threshold in each of these subscales. Individuals in a serious relationship also showed elevated scores in cynicism and total burnout (30.43% for both), but lower levels of emotional exhaustion (17.39%). In terms of professional inefficacy, those in a relationship showed slightly higher prevalence (30.43%) compared to their single, divorced, or widowed counterparts (28.57%). Notably, married individuals had the lowest prevalence of total burnout (20.18%), cynicism (16.51%), and professional inefficacy (21.1%), as well as the second-lowest prevalence in the emotional exhaustion subscale (Figure 2).

Results indicated that forensic medicine workers with children in their care exhibited slightly lower levels of overall burnout, with 21.95% experiencing symptoms consistent with burnout compared to 25.35% of those without children. On the cynicism and professional inefficacy subscales, the results were nearly identical between the two groups, with prevalence of 20.73% vs. 21.12% for cynicism and 23.17% vs. 23.94% for professional inefficacy. However, a different pattern emerged on the emotional exhaustion subscale, where workers with children reported higher levels of exhaustion (25.6%) compared to those without children (19.71%) (Figure 2).

Results showed that total burnout scores were highest among forensic medicine residents, with 27.27% experiencing burnout, followed by forensic medicine doctors at 23.72%. Residents also had the highest prevalence on the cynicism subscale, with 27.27% scoring above the cut-off, followed by morgue staff. In contrast, the emotional exhaustion subscale revealed that forensic medicine doctors had the highest prevalence (27.11%), followed by morgue staff (23.8%). Only 18.18% of medical residents reported high levels of exhaustion during the study period. Professional inefficacy was most prevalent among morgue personnel, with 26.19% scoring above the threshold. They were followed by non-morgue staff such as psychiatrists, geneticists, chemists, biologists, and medical registrars, with 23.33% reporting high inefficacy scores. Notably, this latter group recorded the lowest scores on the other subscales, as well as the lowest overall burnout prevalence (Figure 2).

The questionnaire was completed by forensic medicine personnel from various regions across the country, allowing us to analyze the geographical distribution of burnout. The results indicated that the highest prevalence of total burnout was found in Bucharest, with 37.5% of respondents exceeding the cut-off value, followed by the Dobrogea and Muntenia regions, both with a prevalence of 31.25%. In contrast, the lowest prevalence was observed among workers from the Banat–Crișana–Maramureș region, where only 16.66% reported symptoms of burnout. A similar regional distribution pattern was observed for cynicism, with the highest scores again recorded in Bucharest (37.5%) and the Muntenia region (25%). The Oltenia region registered the lowest levels of cynicism and professional inefficacy among all the regions studied. In contrast, emotional exhaustion showed a different pattern. The lowest exhaustion prevalence was reported in Bucharest (12.5%), while the highest was recorded in the Muntenia region (37.5%), followed by the Oltenia region (27.27%) (Figure 2).

**Figure 3:**
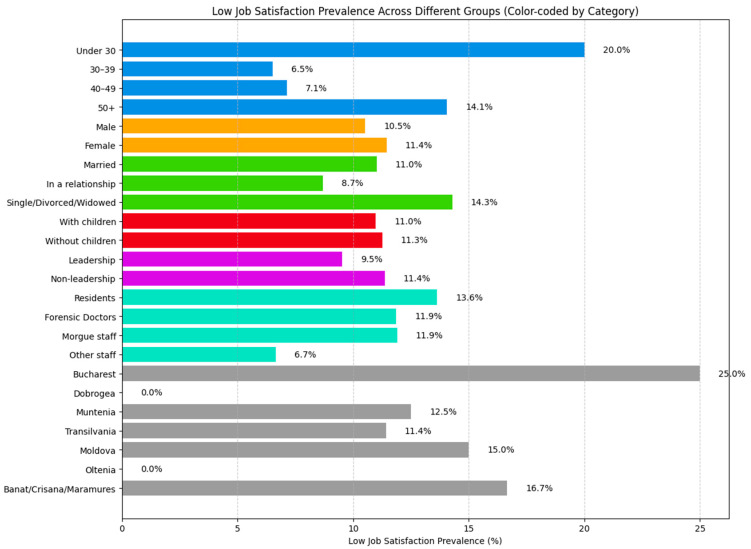
Low-job-satisfaction prevalence across different groups.

Forensic medicine professionals holding leadership positions reported lower total burnout scores and lower levels of cynicism and professional inefficacy compared to employees without such roles. Among workers without a leadership position, 25% reported feeling burned out and inefficient at work, while 22.72% scored above the cut-off for cynicism. In contrast, chief doctors and nurses exhibited notably lower scores on these subscales, with 14.28% reporting high professional inefficacy and only 9.52% showing elevated levels of cynicism. Total burnout was also less prevalent in this group, affecting only 14.28% of individuals in leadership roles. However, a different trend emerged on the emotional exhaustion subscale, where professionals in leadership positions reported a slightly higher prevalence of exhaustion (23.8%) compared to their non-leadership counterparts (22.72%) (Figure 3).

Based on the JSS results, the research group was divided into three categories: individuals with low scores, indicating job dissatisfaction; those with high scores, indicating high job satisfaction; and a middle category, representing participants who were ambivalent about their level of job satisfaction.

The majority of forensic medicine workers reported being satisfied with their jobs, accounting for 51.63% of the total cohort. Only 11.11% of respondents expressed dissatisfaction, while the remaining participants were ambivalent regarding their job satisfaction.

Gender analysis revealed that female employees were slightly more dissatisfied (11.45%) compared to male employees (10.52%). However, job satisfaction was notably higher among men, with 57.89% reporting high satisfaction compared to 47.91% of women (Figure 3).

Similarly to the patterns observed in burnout, lower job satisfaction was more prevalent among younger forensic professionals, with 20% of individuals under 30 years reporting dissatisfaction. In contrast, the 30–39 age group reported the highest levels of satisfaction, with 63.04% expressing contentment with their current position (Figure 3).

No significant differences in job satisfaction were observed between employees with or without children in their care.

However, single, divorced, or widowed individuals reported slightly higher dissatisfaction (14.28%) compared to married individuals (11%). The most satisfied group appeared to be workers in a committed relationship, 60.86% of whom reported high job satisfaction (Figure 3).

In terms of professional roles, forensic medicine residents reported the highest levels of job dissatisfaction (13.63%), while staff members not directly involved with morgue activities (such as biologists, chemists, registrars, or psychiatrists) had the highest job satisfaction, with 63.33% expressing contentment (Figure 3).

Geographic differences were also noted: 25% of forensic medicine workers in Bucharest reported job dissatisfaction, whereas 72.72% of workers from the Oltenia region expressed high satisfaction. Lastly, chief doctors and assistants reported higher levels of job satisfaction (52.38%) compared to employees without a leadership role, 11.36% of whom were dissatisfied with their job (Figure 3).

USAQ results revealed that the majority of forensic medicine employees exhibited low or very low levels of unconditional self-acceptance (39.21%), while only 17.64% reported high or very high levels.

**Figure 4:**
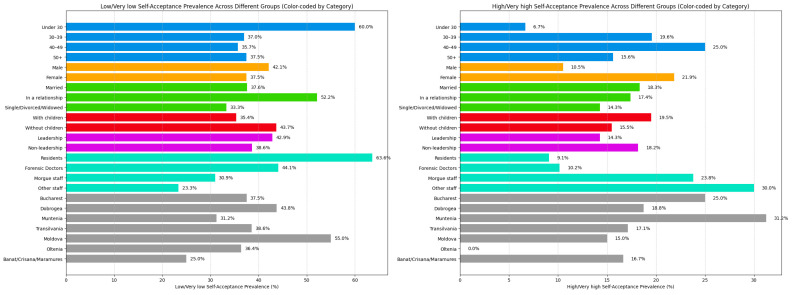
Low/very low and high/very high unconditional self-acceptance prevalence across different groups.

Gender-based analysis showed that men had slightly lower levels of self-acceptance than women, with 42.09% of male participants scoring in the low or very low range compared to 37.49% of females. Conversely, 21.86% of female employees reported high or very high self-acceptance, while only 10.52% of men scored high, and none scored very high (Figure 4).

Age-based comparisons indicated that younger employees had the lowest levels of self-acceptance, with 60% of those under 30 years scoring lowly or very lowly. In contrast, 25% of participants aged 40–49 years and 13.04% of those aged 30–39 years reported high self-acceptance, with an additional 6.52% of the latter group scoring in the very high range (Figure 4).

Employees with children in their care showed slightly higher self-acceptance, with 19.5% reporting high or very high scores compared to 15.48% among those without children, the latter group also having a higher proportion of low or very low scores (43.66%) (Figure 4).

In terms of marital status, 14.28% of single, divorced, or widowed workers reported very low self-acceptance compared to 7.33% of married workers and 4.34% of those in a relationship. Interestingly, low levels of self-acceptance were most common among individuals in a relationship (47.82%), followed by married individuals (30.27%) and then single/divorced/widowed workers (19.04%). However, high or very high levels of self-acceptance were most frequently observed among married workers (18.33%), while none of the single/divorced/widowed participants scored in the very high range (Figure 4).

Regarding professional roles, forensic medicine residents had the lowest self-acceptance scores, with 22.72% reporting very low and 40.9% low self-acceptance. In contrast, 30% of staff not directly involved with morgue activities reported high or very high unconditional self-acceptance (Figure 4).

Regional differences were also evident: 55% of workers from the Moldova region reported low or very low self-acceptance, 31.25% of those from Muntenia scored high, and 12.5% of employees from Dobrogea reported very high self-acceptance (Figure 4).

In terms of leadership, results were similar between groups. Among employees without leadership positions, 8.33% scored very low and 30.3% low on the USAQ, while among those in leadership roles, 4.76% scored very low and 38.09% low. On the other hand, 18.17% of employees without leadership roles reported high or very high self-acceptance compared to 14.28% of individuals in leadership positions, with none of the latter scoring in the very high range (Figure 4).

Among individuals who reported job dissatisfaction, 58.82% met the criteria for burnout syndrome. Within this group, 52.94% exhibited high emotional exhaustion, 41.17% scored high on cynicism, and 64.7% reported high levels of professional inefficacy. In contrast, among those who were satisfied with their job, only 8.86% met the criteria for burnout. Furthermore, 11.39% reported high emotional exhaustion, 8.86% scored high on cynicism, and only 7.59% exhibited high professional inefficacy.

Comparison of burnout levels with USAQ scores revealed that 47.21% of individuals with high total burnout scores exhibited low or very low unconditional self-acceptance. Similar patterns were observed across the subscales: 48.57% of individuals with high emotional exhaustion, 46.87% of those with high cynicism, and 44.43% of those experiencing high professional inefficacy also reported low or very low self-acceptance. In contrast, only 5.54% of individuals with high total burnout scores reported high or very high self-acceptance. The same trend was evident across the subscales: just 2.85% of workers with high exhaustion, 6.24% of those with high cynicism, and 8.32% of those with high professional inefficacy demonstrated high or very high levels of unconditional self-acceptance.

### 3.2. Mediation Analysis

All path estimates were statistically significant: the one from job satisfaction to un-conditional acceptance (path a: b = 0.119, SE = 0.03 *p* < 0.001, 95% CI [0.04; 0.19]), from un-conditional acceptance to burnout (path b: b = −0.271, SE = 0.08 *p* < 0.001, 95% CI [−0.41; −0.12]), and the direct path from job satisfaction to burnout (direct effect: b = −0.032, SE = 0.01, *p* = 0.019, 95% CI [−0.59; −0.24]). The mediation effect was statistically significant (indirect effect: b = 0.119, SE = 0.02, *p* = 0.001, 95% CI [−0.06; −0.01]), suggesting unconditional acceptance mediates 10% of the total effect from job satisfaction to burnout symptoms (Figure 4). The mediation model explained 40.8% of the variance in burnout (R^2^ = 0.408), indicating that job satisfaction and unconditional self-acceptance together substantially predict burnout. Job satisfaction explained 6.8% of the variance in unconditional self-acceptance (R^2^ = 0.068), suggesting a modest association with the mediator (see Table 3).

**Figure 5:**
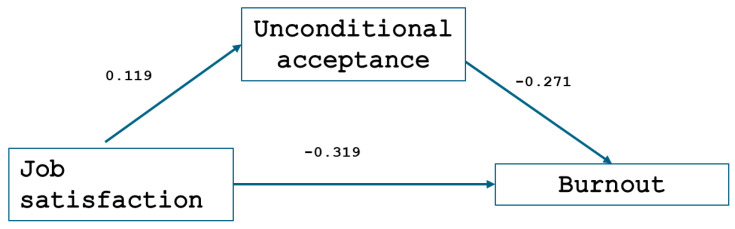
Mediation paths between job satisfaction, burnout, and unconditional self-acceptance in forensic medicine personnel.

In addition to the mediation analysis, a multiple regression analysis was conducted to identify independent predictors of burnout. The regression model included job satisfaction and unconditional self-acceptance as predictors. The results showed that job satisfaction (β = −0.549, *p* < 0.001) and unconditional self-acceptance (β = −0.213, *p* = 0.001) were significant negative predictors of burnout. The model explained 40.8% (R^2^ 0.408) of the variance in burnout, suggesting that both job satisfaction and unconditional self-acceptance contribute uniquely to burnout levels (see Figure 5).

In the current study, a post hoc power analysis was conducted using MedPower (https://davidakenny.shinyapps.io/MedPower/, accessed on 5 May 2025) to assess the statistical power for the mediation model. The analysis revealed that the path from unconditional acceptance to burnout (path b) demonstrated high statistical power (0.926), suggesting a reliable detection of this effect. However, the total effect (path c) and the direct effect (path c′) showed lower power values (0.123 and 0.069, respectively), indicating that subtle effects may not have been adequately detected in the study. These results highlight the need for larger samples in future research or a priori power analysis to ensure that all effects are reliably identified.

## 4. Discussion

Overall, results from the MBI-GS indicate that approximately a quarter of forensic medicine employees in Romania experience burnout syndrome. Similar prevalence rates were observed for both emotional exhaustion and professional inefficacy within this professional group. In contrast, cynicism was slightly less prevalent, with about a fifth of employees scoring above the threshold. These findings closely mirror those of our recent single-center, multiphase study on burnout in forensic medicine, where during the first phase at the beginning of the COVID-19 pandemic, moderate burnout levels were reported in approximately 24% of participants. This was followed by a sharp increase to just over 50% during the mid-pandemic period, before regressing to levels comparable to the pre-pandemic phase [35].

One small study concluded that forensic medicine workers exhibited low levels of total burnout with low emotional exhaustion and moderate levels of emotional detachment and professional inefficacy under normal working conditions [36]. Similar findings were reported by Iorga et al. [26]. Other studies have revealed high levels of emotional exhaustion (EE), moderate depersonalization (DP), and low personal accomplishment (PA) in forensic settings [20]. In particular, 40.5% of forensic medicine professionals who dealt with highly distressing cases—such as those involving young child victims of sexual assault, murder, suicide, aggressive detainees, or decomposed bodies—reported high levels of DP, followed by 25% experiencing high EE and 20.2% showing low PA [16]. In another study involving morgue personnel, including autopsy technicians, forensic specialists, and residents, 76% of subjects reported burnout in the PA domain, while 32% were emotionally exhausted and 14% experienced depersonalization [17].

Occupational stressors appear to have the greatest impact on the development of burnout. Studies have shown that limited resources and organizational issues are strongly associated with increased emotional exhaustion, while organizational dysfunction and interpersonal conflicts among staff negatively affect professional inefficacy [20]. Additionally, high emotional job demands, insufficient financial compensation, and poor communication from supervisors have been identified as important burnout triggers [16]. In the morgue setting, exposure to traumatic or disturbing cases can serve as a catalyst for burnout syndrome. Forensic practitioners frequently reported that cases involving pregnant women, infant victims, burned or fragmented corpses, and decomposed bodies were psychologically overwhelming [17]. Furthermore, high burnout prevalence has been linked to certain personality traits, particularly neuroticism, as well as to low job satisfaction. Individuals experiencing burnout often reported symptoms of depression and various physical health concerns, reinforcing the complex psychological and physiological dimensions of the syndrome [37].

When comparing burnout levels in forensic medicine with those in other frontline and high-stress medical specialties, notable differences emerge. For instance, emergency department workers experience occupational burnout at a rate of approximately 80%, with 7% classified as having severe burnout. Within this group, 68% report emotional exhaustion, 53% experience depersonalization, and 28% show low personal accomplishment [38]. These elevated levels of burnout among emergency department personnel have been strongly associated with the high frequency of night shifts and direct exposure to workplace violence, including both verbal and physical aggression from patients and their families [38]. While comparing burnout rates across medical specialties may offer context, caution is warranted due to variations in measurement tools, scoring thresholds, and institutional settings. Comparisons with emergency department personnel should be interpreted in light of different burnout instruments and cut-off criteria used in other studies. Additionally, our study did not measure certain contextual factors, such as organizational culture, administrative burden, or case complexity, that may have contributed to burnout, but were outside the scope of this investigation. Future research should incorporate such variables to better understand institutional drivers of psychological distress.

As previously highlighted, acute stressful situations, such as the COVID-19 pandemic, serve as significant triggers for the development of burnout syndrome. One study conducted during the pandemic found that 34.2% of frontline health professionals experienced emotional exhaustion, 50.8% scored high on cynicism, and 35.2% reported low professional efficacy [39].

With regard to gender, the current results indicate that male forensic workers show a slightly higher risk of total burnout, as well as emotional exhaustion and professional inefficacy. Female respondents, on the other hand, showed a higher level of cynicism. Several studies involving forensic medicine professionals have found no significant associations between gender and burnout [17,40]. However, some findings suggest that female forensic practitioners tend to exhibit lower professional efficacy [22] and may be more susceptible to distress intolerance, depression, and PTSD [41]. In broader healthcare settings, studies have shown that female professionals working in death-related fields, such as forensics, intensive care units, emergency departments, and palliative care, are more prone to emotional exhaustion and depersonalization, primarily due to dual responsibilities at work and home, societal expectations, and insufficient institutional support [42]. Other studies have similarly reported that female healthcare workers experience higher levels of emotional exhaustion, which stands in contrast to the findings of our current study [43,44,45,46].

Our results suggest that younger individuals are more prone to developing burnout, including higher levels of exhaustion, cynicism, and professional inefficacy. Similar findings have been reported in other studies [42,47,48,49]. In contrast, older professionals tend to report lower emotional exhaustion, less cynicism, and higher professional efficacy, suggesting that experience, improved working conditions, and adaptation over time may serve as protective factors against burnout [48]. This trend may also be explained by evidence that older healthcare workers engage less frequently in negative coping strategies, which are known to exacerbate burnout symptoms [49]. Furthermore, recent research has shown that burnout can begin as early as medical school, with approximately half of students meeting the criteria for burnout syndrome [40].

Our analysis revealed that married forensic medicine workers are at lower risk of developing burnout, particularly in relation to cynicism and professional inefficacy, though this protective effect was not observed for emotional exhaustion.

Additionally, individuals with children in their care showed a slightly lower likelihood of experiencing burnout and cynicism, but appeared to be at greater risk of emotional exhaustion. Similar findings have been reported in the literature, which also highlights the protective role of marriage and parenthood in relation to certain dimensions of burnout [38,50,51].

Among professional groups, forensic medicine residents were found to have a slightly higher risk of developing burnout, cynicism, and professional inefficacy, although emotional exhaustion was more prevalent among specialist and primary forensic medicine doctors. The existing literature presents ambivalent findings in this regard. Some studies suggest that autopsy technicians are more likely to experience higher levels of exhaustion than medical doctors [37]. Other studies, however, support our current findings, showing that doctors are more likely to report elevated burnout levels, particularly in exhaustion and depersonalization [45,46,52,53]. In contrast, several studies have indicated that nurses are more frequently affected by emotional exhaustion and depersonalization [44,51,54].

Working in the capital appears to be associated with higher levels of burnout, particularly in relation to cynicism and reduced professional efficacy. Similar patterns were observed in the Muntenia region, where elevated levels of emotional exhaustion were also noted.

Conversely, holding a leadership position tends to be protective against burnout, cynicism, and professional inefficacy, although individuals in such roles may experience slightly higher levels of exhaustion. These findings are consistent with prior research indicating that supervisors in medical settings report significantly lower emotional exhaustion and depersonalization than other staff members [55]. Similarly, head nurses exhibit lower levels of exhaustion and depersonalization compared to other senior nurses [56]. Additional studies confirm that head doctors tend to have significantly lower exhaustion and cynicism scores than other healthcare personnel [57].

In regard to job satisfaction, our analysis revealed that the majority of forensic medicine professionals are satisfied with their current position. Female workers tend to report slightly higher levels of job dissatisfaction, whereas male professionals are more likely to express satisfaction with their roles. Having children in one’s care did not appear to significantly influence job satisfaction. However, individuals who were single, divorced, or widowed reported greater dissatisfaction compared to those in a relationship, the latter group showing the highest prevalence of job satisfaction. Notably, high levels of dissatisfaction were observed among forensic workers under the age of 30 and among forensic medicine residents, while greater satisfaction was found among those in the 30–39 age group and among personnel without direct contact with the morgue. Regionally, higher dissatisfaction rates were recorded in the capital city, Bucharest, whereas the Oltenia region reported a higher prevalence of job satisfaction. Finally, individuals in leadership positions, such as chief doctors and head nurses, showed greater job satisfaction compared to their subordinates.

Memarian et al. found similar results, reporting high levels of job satisfaction among forensic medicine specialists, with levels comparable to those of internists and pediatricians. However, they observed no significant differences in job satisfaction based on gender, age, or work experience, although dissatisfaction was associated with poor communication and organizational structure within forensic departments [58]. Two other studies conducted in Romania on forensic physicians also reported high overall job satisfaction. Additionally, job satisfaction was found to be negatively correlated with emotional exhaustion and depersonalization and positively correlated with support from colleagues and higher levels of personal accomplishment [26,59].

High levels of job satisfaction were also observed among frontline medical staff, even during the COVID-19 pandemic. The study indicated that greater work experience, longer sleep duration, and voluntary participation in COVID-19-related efforts were positively associated with job satisfaction. In contrast, individuals with higher educational levels and those who were engaged in COVID-19-related duties for extended periods reported higher levels of dissatisfaction [60]. Similarly, Kabbasch et al. examined job satisfaction among physicians and found that 23.8% of secondary care physicians were dissatisfied compared to only 5.9% of those in tertiary care. Dissatisfaction was primarily linked to work burden, limited promotion opportunities, financial concerns, and poor supervision [61].

Job satisfaction has been found to be strongly associated with work–life balance, organizational commitment, and employee performance. In contrast, dissatisfaction is frequently driven by factors such as inadequate salary, limited opportunities for promotion, lack of management support, poor physical working conditions, and the absence of recognition or autonomy [62].

Almost 50% of forensic medicine workers reported low or very low levels of self-acceptance, while only approximately 20% demonstrated high or very high self-acceptance. Male professionals and those without children in their care tended to exhibit lower levels of self-acceptance. Similarly to the trends observed in burnout and job satisfaction, more than half of individuals under the age of 30, as well as more than half of forensic medicine residents, reported low or very low self-acceptance. Interestingly, chief doctors and nurses exhibited slightly lower self-acceptance than their subordinates.

Mediation analysis revealed that low job satisfaction may act as a generator of burnout syndrome, while unconditional self-acceptance can serve as a mediator that reduces burnout symptoms among forensic medicine professionals. The mediating role of self-acceptance was also examined by Zhang et al. in a study involving medical students. Their results showed that self-acceptance, along with interpersonal adaptation, mediated the impact of regulatory emotional self-efficacy on psychological distress [63]. Similarly, a study involving 813 psychiatric nurses found that self-acceptance contributes to the development of positive coping strategies [64]. The mediation analysis demonstrated that unconditional self-acceptance accounted for approximately 10% of the relationship between job satisfaction and burnout. While this indirect effect was statistically significant, its magnitude is relatively small. This finding aligns with the understanding that burnout is a multifactorial phenomenon influenced by a wide range of occupational, psychological, and organizational variables. The modest effect size of self-acceptance may reflect its role as one of several potential protective factors rather than a dominant explanatory mechanism. Nonetheless, even small effects can hold practical significance, particularly when they involve modifiable traits that may be enhanced through targeted interventions. This result highlights the need for future research to explore additional mediators that may more fully explain the pathway between job dissatisfaction and burnout in forensic medicine professionals.

## 5. Conclusions

This national study provides a comprehensive overview of burnout syndrome among forensic medicine professionals in Romania, revealing that approximately a quarter of participants experience high levels of emotional exhaustion, professional inefficacy, and total burnout, while cynicism was present in a fifth of the cohort. Burnout prevalence was notably higher among younger professionals, especially those under the age of 30 and forensic medicine residents, while lower rates were observed among those in leadership positions and professionals in long-term partnerships or with children in their care.

Over half of the forensic workers were satisfied with their job, with dissatisfaction being more common among females, younger individuals, and residents. High satisfaction rates were observed in workers aged 30–39, those in a relationship, and staff members not directly exposed to morgue-related activities. Interestingly, leadership positions were associated with higher job satisfaction, and regional disparities emerged, with the capital city exhibiting the highest dissatisfaction levels.

Nearly half of the participants reported low or very low levels of self-acceptance, particularly among males, workers without children, and younger staff members. Only about a fifth of the respondents demonstrated high or very high self-acceptance. These findings suggest that emotional self-evaluation and internal coping mechanisms may play a critical role in occupational well-being.

Furthermore, mediation analysis demonstrated that low job satisfaction significantly contributed to burnout, particularly emotional exhaustion and professional inefficacy. However, unconditional self-acceptance was found to partially mediate this relationship, suggesting that internal emotional resilience may buffer the impact of workplace dissatisfaction.

## 6. Clinical Implications and Future Research Directions

Given the high prevalence of burnout, particularly among younger professionals and residents, there is an urgent need for structured mental health support within forensic medicine. Regular psychological screening, access to emotional support services, and targeted interventions, such as resilience training and self-acceptance enhancement, should be prioritized. Organizational strategies to improve job satisfaction, including workload management, supportive leadership, and fair promotion pathways, are essential. Tailored approaches that consider regional differences and specific professional roles may further enhance the effectiveness of burnout prevention and staff well-being initiatives. Also, in order to address burnout in forensic medicine specifically, we suggest concrete, evidence-based interventions beyond individual-level psychological training. These include the implementation of structured specific peer-support programs for employees working in a morgue setting, increased access to professional supervision, improved scheduling to avoid overwork, and redistribution of emotionally demanding tasks where possible. Organization-level efforts that enhance communication, recognition, and autonomy may also play a protective role. Embedding such strategies within forensic departments could offer more sustainable burnout prevention than generic resilience interventions alone. The findings of this study also emphasize the significant role of job satisfaction and unconditional self-acceptance in the development of burnout among forensic medicine personnel. Future research should aim to replicate these findings using longitudinal designs to better establish causal relationships. There is a need for additional studies evaluating the effectiveness of targeted interventions such as self-acceptance training, mindfulness programs, or job restructuring efforts. Expanding research to include other psychological variables and even a more diverse forensic population may also give deeper insights into protective mechanisms against burnout in this field.

## 7. Limitations

Despite the strength of this study in capturing burnout and psychosocial indicators in a national cohort of forensic medicine professionals, several limitations must be acknowledged. First, the study employed a cross-sectional design, which limits the ability to infer causal relationships between variables such as burnout, job satisfaction, and self-acceptance.

Although the mediation analysis revealed statistically significant relationships among job satisfaction, unconditional self-acceptance, and burnout, the cross-sectional design of this study limits causal interpretation. Mediation assumes a temporal sequence between predictor, mediator, and outcome; however, with cross-sectional data, this sequence cannot be empirically verified. Therefore, the observed associations should be interpreted with caution, and future longitudinal studies are needed to confirm the directionality of these relationships. Second, all data were collected through self-reported questionnaires, which may be subject to recall bias, social desirability bias, and subjective interpretation of items. Third, although the study included professionals from multiple regions in Romania, the sample was not evenly distributed geographically, with a higher concentration of responses from certain areas (e.g., Transylvania), potentially affecting generalizability.

Another limitation of this study is the absence of a confirmatory factor analysis (CFA) for the psychometric validation of the instruments used. Although we employed well-established scales with previously validated factor structures, including the MBI-GS, JSS, and USAQ, we did not conduct CFA within our own sample due to resource constraints.

Moreover, the response rate from specific professional subgroups, such as autopsy technicians, drivers, and auxiliary staff, was low, limiting the robustness of subgroup comparisons. Additionally, although widely used and validated tools (MBI-GS, JSS, USAQ) were applied, cut-off values were established using percentile-based internal calculations, which may not align with international standards or allow for direct comparison with other studies. The absence of longitudinal follow-up also precludes evaluation of burnout progression or the long-term impact of protective or risk factors.

Other potentially influential variables, such as workload intensity, institutional support systems, previous psychological diagnoses, coping styles, and exposure to traumatic cases, were not measured in depth and could further contextualize the results. Finally, while the study included questions on leadership roles, it did not explore organizational culture, managerial practices, or team dynamics, all of which may influence job satisfaction and burnout.

## Figures and Tables

**Table 1:** Descriptive analysis of burnout syndrome, job satisfaction and unconditional self-acceptance in forensic medicine workers.

	Burnout	Job Satisfaction	USAQ
N	153	153	153
Median	20.000	146.000	86.000
Mean	22.791	143.987	87.503
Standard Deviation	16.133	27.788	12.673
Skewness	0.931	−0.267	0.097
Standard Error of Skewness	0.196	0.196	0.196
Minimum	0.000	67.000	56.000
Maximum	90.000	206.000	122.000

Note: N = sample size; USAQ = Unconditional Self-Acceptance Questionnaire.

**Table 2:** Correlation matrix using Pearson correlation coefficients.

	Burnout	Job Satisfaction	USAQ
Burnout	-		
Job Satisfaction	−0.60 **	-	
USAQ	−0.35 **	0.26 **	-

Note: ** *p* = 0.001; USAQ = Unconditional Self-Acceptance Questionnaire.

**Table 3:** Summary of mediation model path estimates and variance explained.

Path	b	SE	95% CI	*p*-Value
Job satisfaction → unconditional acceptance (a)	0.119	0.03	[0.04, 0.19]	<0.001
Unconditional acceptance → burnout (b)	−0.271	0.08	[−0.41, −0.12]	<0.001
Job satisfaction → burnout (direct effect, c′)	−0.032	0.01	[−0.59, −0.24]	0.019
Indirect effect (a × b)	0.119	0.02	[−0.06, −0.01]	0.001
Total variance explained in burnout (R^2^)	–	–	–	0.408
Variance explained in mediator (R^2^)	–	–	–	0.068

## Data Availability

The raw data supporting the conclusions of this article will be made available by the authors upon request.

## Abbreviations

The following abbreviations are used in this manuscript:

MBI	Maslach Burnout Inventory
MBI-GS	Maslach Burnout Inventory—General Survey
JSS	Job Satisfaction Survey
USAQ	Unconditional Self-Acceptance Questionnaire
JD-R	Job Demands–Resources
